# Interactions between polyols and wheat biopolymers in a bread model system fortified with inulin: A Fourier transform infrared study

**DOI:** 10.1016/j.heliyon.2018.e01017

**Published:** 2018-12-08

**Authors:** Amir Pourfarzad, Zahra Ahmadian, Mohammad B. Habibi-Najafi

**Affiliations:** a Department of Food Science and Technology, Faculty of Agricultural Sciences, University of Guilan, Rasht, Iran; b Department of Food Processing, Research Institute of Food Science and Technology (RIFST), Mashhad, Iran; c Department of Food Science and Technology, Faculty of Agriculture, Ferdowsi University of Mashhad, Mashhad, Iran

**Keywords:** Food science, Natural product chemistry, Analytical chemistry, Food technology, Food analysis

## Abstract

One of the ways to improve food safety and reduce community health risks is fortification of these products with inulin. Inulin, in spite of the effects and nutritional benefits, will also have undesirable effects on the quality and shelf life of bread. In this study, the interactions between polyols as improvers (i.e. glycerol, sorbitol and propylene glycol) and major biopolymers of wheat flour (i.e. starch and gluten) were examined in model systems fortified with Serish inulin by Fourier transform infrared (FTIR) spectroscopy. The changes in starch structure were estimated focusing on the ratios of the heights of the bands at 1047 and 1022 cm^−1^ which expresses the quantity of ordered starch to amorphous starch. At first and 5th days of storage, this ratio of control sample was higher than polyol treated samples. It was proved from Gaussian–Lorenzian curve fitting that the relative contribution of characteristic peaks of β-turns and intramolecular β-sheets was consecutively increased when polyol proportion of models increased. Whereas, content of intermolecular β-sheets and α-helix was slightly decreased with increasing of polyols in the models. Briefly, polyols especially 5% propylene glycol, could be used to reduce the undesirable effects of inulin on the quality parameters of dough and bread.

## 1 Introduction

Functional foods have shown a high potential of growth in the recent years. In this regard, the enrichment of bread with the functional components such as inulin, is considered an exciting subject to both the consumer as well as the cereal industry, since wheat bread is an important food product and largely consumed all over the world as staple food [1].

Inulin is a natural part of several edible fruits and vegetables. It is classified as neutral polysaccharides as it is containing oligomeric and polymeric sequences of fructose with a glucose at the end of the chain connected to the last fructose part. It improves systemic health by reduction of disease risks such as intestinal infections, irritable bowel diseases, colon cancer, osteoporosis and obesity [2]. In addition to its interesting nutritional and health benefit properties, inulin is also used in food formulations for its techno-functional properties such as fat substitute, bulk agent and water retention [3, 4]. Serish (*Eremurus spectabilis*) is a part of the Liliaceae family and geographically scattered throughout the area of South and Central Asia. It could be used as a new resource of inulin as its roots gather high amounts of fructans during their growth [5].

Bread is created from molecular scattering of biopolymers and their complexes. As the complexed molecules gain new properties compared with the individual, the functional properties of dough and bread reveal the physico-chemical properties of both the individual and the complexed macromolecules. Interactions between macromolecules contribute to the variety of bread structures because the complexed molecules achieve new properties in comparison with the individual. The physico-chemical properties of both the complexed and the individual macromolecules affect the functional properties of bread. Thus, monitoring of complex-formation and interactions between macromolecules could contribute to an increased control over the processes that determine quality and shelf life [6]. Some important interactions, which have potential effect on bread quality and shelf life, are breakage and formation of both covalent and non-covalent bonds in wheat proteins during dough mixing [7], cross-linking and polymerization structural of gluten polymers during baking [8], starch gelatinization, pasting and retrogradation through baking [9], Interactions between gelatinized starch granules and the gluten network during baking [10].

Polyols are one of the extensively used additives in the baking industry. They lower the water activity and improve texture and mouthfeel. Polyols have been used effectively to increase the shelf life of flat bread [11], flour tortillas [12] and meal bread [13]. Because of the great variation in the effects promoted by the different polyols, a systematic study on the influence of polyols on the conformations of protein/polysaccharides seems to be necessary.

FTIR spectroscopy is a rapid, versatile, and sensitive tool that has been played a pioneering role in elucidating the structure, physical properties and interactions of heterogeneous foods and biological materials. It can provide valuable information about the development process of perfect formulations and process parameters [14]. IR spectroscopy is based on the conversion of IR radiation into molecular vibrations. FTIR spectroscopy can be applied to consider the secondary structures and conformations of protein/polysaccharides obtained from the characteristic absorption bands of unique functional groups included within these biopolymers [15]. Determination of protein secondary structure using FTIR spectroscopy has numerous advantages for wheat dough systems. First, sample status is not an issue. Dough systems are clearly not crystallized and are extremely concentrated. Thus, they are inappropriate for traditional structural characterization techniques such as x-ray crystallography and circular dichroism spectroscopy. These methods need dry, crystallized proteins or optically clear solutions. In addition, sample measurement by FTIR is rapid and non-destructive. Finally, no sample preparation is necessary. Samples can be examined directly from the mixer that restrains the existence of artifacts from sample preparation and the time lapse between the experimental treatment and measurement [16].

Previous studies showed that added inulin negatively influences the properties of bread doughs and subsequently breads [17, 18, 19, 20]. To our knowledge, no reports have been found on the investigation of the interactions between added inulin, typical bread ingredients and polyols, which may then lead to the advance of innovative functional breads with high quality and shelf life. Thus, the present study is considered the first effort aiming: (a) to investigate the conformational changes of systems containing Serish inulin, wheat biopolymers (gluten and starch) and polyols (glycerol, sorbitol and propylene glycol) in model systems using FT-IR technique; (b) to explore the secondary structure changes of gluten in model systems caused by polyols using second-derivative transformation and Gaussian–Lorenzian curve fitting.

## 2 Materials and methods

### 2.1 Materials

The Serish root powders were attained from the local medical plant market, Mashhad, Iran. Wheat gluten (catalog number G-5004, ≥80% protein) and wheat starch (catalog number G-5004, ≥98% starch) were procured from Sigma–Aldrich (Gillingham, Dorset, UK). Propylene glycol was purchased from J.T. Baker Chemical Co., Phillipsburg, NJ, USA. Glycerol (Superol glycerin USP) was purchased from Proctor and Gamble Co., Cincinnati, OH, USA. Sorbitol (Neosorb, 70 g/100 g solids content) was purchased from Roquette Corporation, Gurnee, IL, USA. All other chemicals, reagents and solvents were of analytical grade.

### 2.2 Methodology

#### 2.2.1 Preparation of Serish inulin

Inulin was extracted from Serish root powder following the method of Pourfarzad et al. [21]. The suspension was then purified, concentrated and precipitated by ethanol [22]. Serish inulin was freeze dried and milled to pass through a 1.0 mm mesh sieve. Dried inulin was kept in hermetically sealed container until used [5]. The characteristics of produced inulin were specified according to Pourfarzad et al. [22]. Its purity and degree of polymerization were 93.8% and 13, respectively.

#### 2.2.2 Model systems preparation

The compositions of model systems consisting of inulin, starch, gluten and polyols examined in this study are given in Table 1. The weight ratios of components in models were chosen according to their ratio in the wheat flour. Level of inulin addition in models was 10% of the sum quantity of other components (i.e. gluten and starch) in accordance with the literature. Polyols were also used at 1 and 5% of overall weight of gluten + starch + inulin [11]. The weight ratio of water to total solids content in the blends was 3:1. Initially, the blends were dispersed in distilled water under continuous agitation. The dispersions were then held at 30 °C for 16 h to permit the components of model systems to interact entirely with each other. The resulting blends were freeze-dried and pulverized under liquid nitrogen to provide fine powders that were used to achieve the test spectra. Inulin, starch, gluten and polyols were each treated individually, to prepare control spectra of the components alone.

**Table 1:** Variables and weight ratios used in model systems.

Treatment	Level	Inulin	Gluten	Starch	Glycerol	Sorbitol	Propylene glycol
Control	-	0.8	1	7	0	0	0
Glycerol	1%	0.8	1	7	0.0088	0	0
	5%	0.8	1	7	0.044	0	0
Sorbitol	1%	0.8	1	7	0	0.0088	0
	5%	0.8	1	7	0	0.044	0
Propylene glycol	1%	0.8	1	7	0	0	0.0088
	5%	0.8	1	7	0	0	0.044

The weight ratios of components in models were selected according to the their ratio in the wheat flour; Level of inulin addition was 10% of total amount of other components according to the literature.

#### 2.2.3 FT-IR analysis

Fourier-transform infrared spectroscopic (FT-IR) studies were performed using a spectrophotometer (Paragon 1000, Perkin Elmer, USA) and spectrum 10 software (Perkin Elmer, USA). Samples were blended with KBr powder and pressed to form tablets before measurement. Spectra were achieved at 4 cm^−1^ of resolution from 4000 cm^−1^–400 cm^−1^. The interference of water and CO_2_ from air was subtracted during scanning [23].

Most IR absorption bands are wide and comprise overlapping parts. Hence, vector normalized second derivatives of average spectra of the spectral divisions were applied for comparisons of spectral variations. Second derivative resolution enhancement was used to thin out the width of infrared bands and increase the division of the overlapping components with Unscrambler v. 9.2 software (Camo process AS, Oslo, Norway). The resolution improvement resulting from the second derivative was such that the number and the position of the bands to be fitted were readily determined. In experiments involving inulin, starch and polyols addition, their spectrum was subtracted from the gluten spectrum. Initially, normalized spectra were subjected to a multipoint linear base-line correction using the Unscrambler software. The Gaussian–Lorenzian mix function was utilized for curve fitting of the amide I band district (1600–1700 cm^−1^).

Proteins usually fold into complex three-dimensional structures that consist of a variety of domains containing polypeptide segments folded into different types of secondary structure. Each of these conformational proteins contributes to the infrared spectrum. Thus, the detected amide I band contours consist of several composites. They are made of many overlapping component bands that signify different structural elements such as α-helices, β-sheets, turns, and non-ordered or irregular structures. The different amide I regions were assigned to protein secondary structures according to previous reports. Evaluated secondary structures of gluten included the intermolecular β-sheet, α-helix, β-turn and intramolecular β-sheet secondary structure contents of gluten in models which estimated from relative band areas in the spectral sections 1620–1640 cm^−1^, 1650-1660 cm^−1^, 1660-1680 cm^−1^, and 1680-1695 cm^−1^, respectively [24]. The iterative data fitting was performed until an acceptable ‘goodness of fit’ was attained. The relative peak areas (RPAs) of the absorbance bands were stated as “percentage of the area of fitted region” [5].

#### 2.2.4 Statistical analyses

Results are given as the average of three replications. To evaluate the significant differences among different interactions, a complete randomized design of triplicate analyzes of samples was performed using the Minitab 15 (Minitab Inc., State College, PA, USA) software. Duncan's multiple tests were accomplished to investigate the statistical differences between treatments.

## 3 Results and discussion

### 3.1 FT-IR spectra for ingredients

**Fig. 1:**
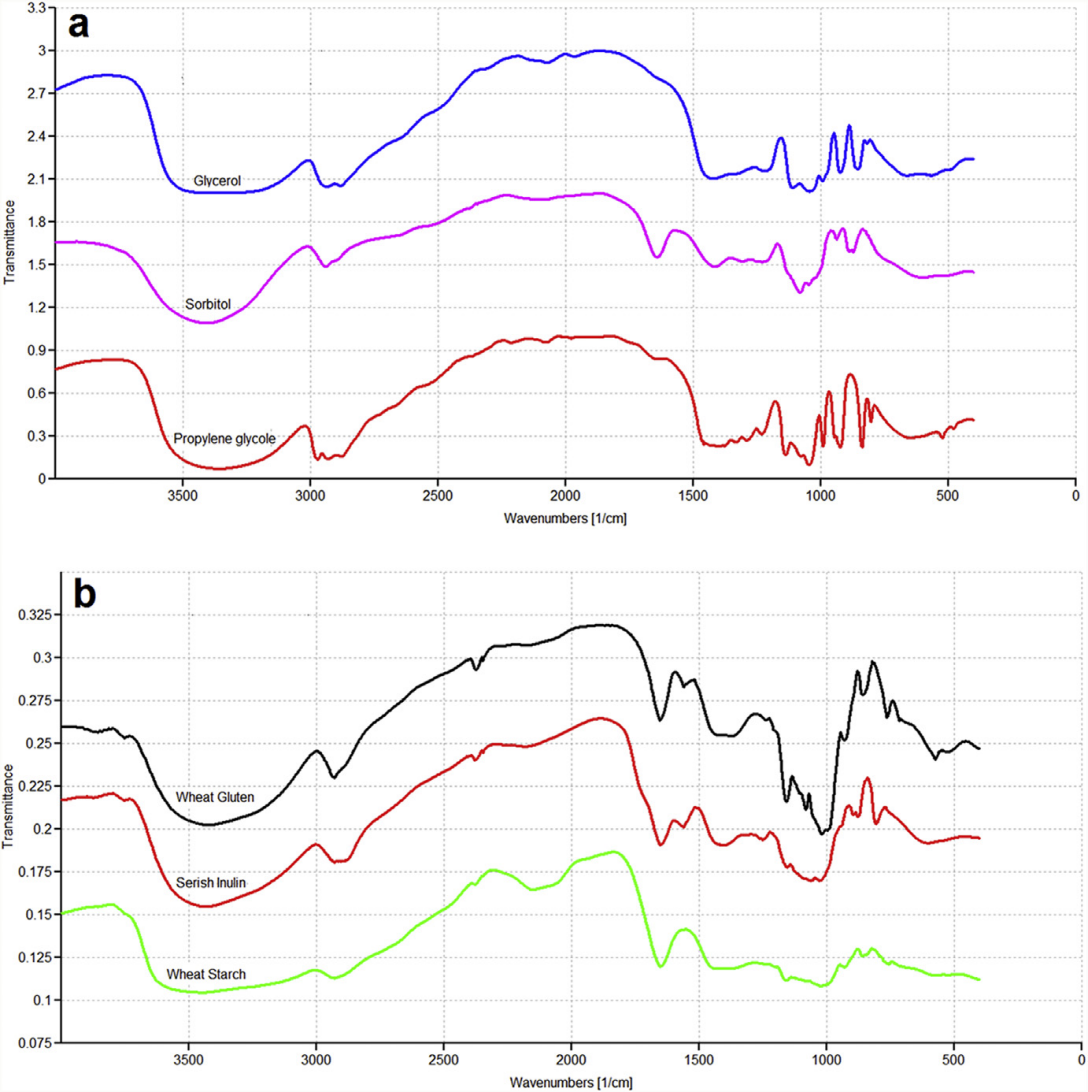
FTIR spectra for components of models; (a) polyols (b) inulin and wheat biopolymers.

FT-IR absorbance spectra for the models components are illustrated in Fig. 1. All ingredients exhibited bands at 2800–3000 cm^−1^ and a wide feature at ∼3300 cm^−1^, as a result of C—H stretching modes and intermolecular H-bonded and O—H stretching modes, respectively [25]. It is evident that polyols have peaks at range 1500–1200 cm^−1^. These peaks are assigned to overlapping of C-H in planes and O-H bending in the polyol molecules. Also, the absorption bands at 2917cm^−1^ was due to the asymmetrical and symmetrical stretching of COH [26]. In the glycerol spectra, the peak intensities at about 1145 and 962 cm^−1^ are responsible for C-O stretching [27]. The key bands of the sorbitol spectra had maximum absorption at 890, 1046, 1084 and 1411 cm^−1^. The bands placed at 1046 and 1084 cm^−1^ were ascribed to C– OH stretching vibrations, whereas the bands with maxima at 890 and 1411 cm^−1^ appeared caused by the in-plane and out-of-plane bending vibrations of O–H bonds, respectively [28]. On the other hand, C–H stretching bands of propylene glycol was also observed at 1442, 1374 cm^−1^ [29]. In the gluten spectrum, CH, and NH or OH stretching modes presented peaks placed at 2934 and 3397 cm^−1^ wavenumbers, respectively. For starch and inulin, further characteristics are observed in the region of 800–1200 cm^−1^ which are distinctive for polysaccharides. The characteristic bands of starch are at 1010, 1080 and 1150 cm^−1^, which are related to the joined C—O and C—C stretching vibrations of the polysaccharide molecules. In the inulin spectrum, the absorbance peaks between 600–800 cm^−1^ exposed the absorption of the C-H aliphatic bending. The relatively strong absorption peak at around 1653 cm^−1^ reproduced the absorption of the OH bending signal of adsorbed water [30]. These results are comparable with that of earlier reports [5].

### 3.2 Effect of polyols on starch

**Fig. 2:**
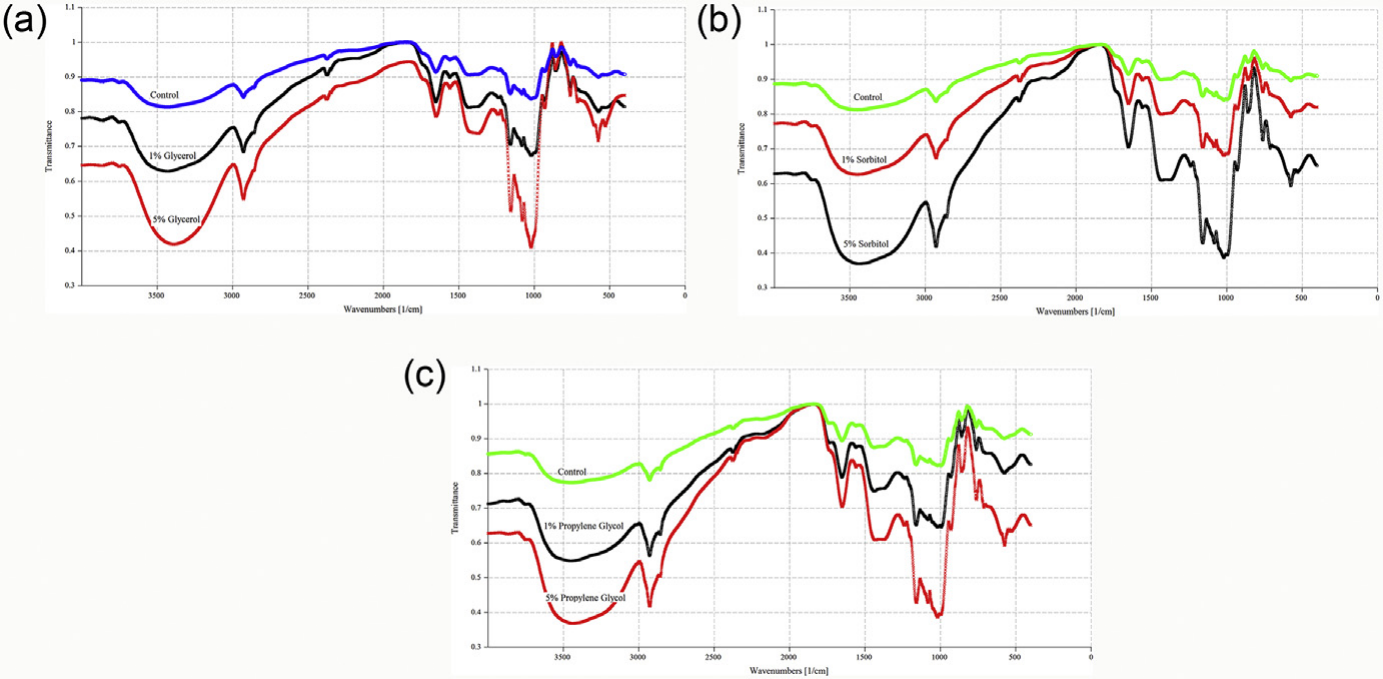
FTIR spectra for models containing glycerol (a), sorbitol (b) and propylene glycol (c) at first day.

The FTIR spectra of models with addition of glycerol, sorbitol and propylene glycol was measured (Fig. 2). The most important peaks in the IR spectra were revealed in two zones, 3700 - 2500 cm^−1^ and 1700–700 cm^−1^. The wide band at 3700–3000 cm^−1^ is an outcome of O–H stretching vibration associated with free, inter, and intra-molecular bound hydroxyl groups. The peaks in the region 3000–2800 cm^−1^ are a consequence of C–H stretching [31]. Different polyols resulted in different extents of absorbance in these regions with the highest from the propylene glycol samples. Furthermore, the intensity of these bonds enhanced by the increase of polyols levels in the system models. This behavior might be due to the enhancement of water entrapped in the model systems because of higher levels of polyols, which was consistent with the increased bands at these wavenumbers. Similarly, intensity of the peaks at 992 and 1653 cm^−1^ was higher than the control and enhanced with increasing of polyols level. These peaks are relevant to hydrogen bonding within the hydroxyl group and are affected to the level of water present [32]. The highest intensity was related to 5% propylene glycol. The model samples with higher intensities within these regions had higher moisture contents. The wavenumbers of highest intensity was also shifted from 3329 to 3357 cm^−1^ by addition of 5% propylene glycol that means more hydroxyl groups of water and polyols were involved in the hydrogen bonds with starch. Starch as a carbohydrate polymer contains extensive hydrogen bonding. Both intramolecular and its interactions with solvent, water or polyols lead to changes in the hydrogen bonding network of the system which may be returned in the FT-IR spectra. Also, the broad band between 1100 and 900 cm^−1^ comes from the stretching vibrations of alcoholic C–O in C–O–H bonds (∼1072 cm^−1^) and in the asymmetric and symmetric C–O–C bonds (∼1018 cm^−1^) [33]. These vibrations are related to the composition of starch and polyols and showed small shifts with addition of the polyols, especially propylene glycol [34]. With addition of propylene glycol, the absorption in these bands was significantly increased indicating the higher interactions between the polymer hydroxyl groups which at high concentrations made various conformational transforms in the polymer [35]. The peak at ∼1650 cm^−1^ (bound water) moved to lower wavenumbers, indicating further water molecules were bound by polyols. Sorbitol is manufactured through hydrogenation of glucose. Its molecule was supposed to present similar to the ring conformations of the glucose molecule [36]. It worked like a plasticizer to reduce the interaction between the starch and inulin polymers. Results show that propylene glycol and glycerol models had the most and the least intense peaks at 1650 cm^−1^, which means that they had the largest and smallest amount of bound water molecules in the models. This result agreed with the moisture determination reported by Pourfarzad et al. [11]. This phenomenon suggests that different etherification reactions between the starch and the polyols occurred.

Fourier transform infrared spectroscopy provides a method to the study of the disorder to order transitions throughout the retrogradation reaction of starch. The IR absorbance band at 1047 cm^−1^ was susceptible to ordered or crystalline structure and the band at 1022 cm^−1^ was related to amorphous structure in starch, and at 995 cm^−1^ which was responsive to water [37]. The ratios of the heights of the bands at 1047 and 1022 cm^−1^, states the quantity of ordered starch to amorphous starch.

In this research, the intensity ratio of 1047 cm^−1^ ⁄ 1022 cm^−1^ can clarify the degree of order in starch (Table 2). At first and 5^th^ days, this ratio of control sample was higher than polyol treated samples. This is caused by the plasticizing influence of the polyols on starch biopolymers [38]. These plasticizers diminish the extent of cross-links in retrograded starch molecules. The polyols' highly hygroscopic nature has been associated in its capability to delay staling by producing an entangled amorphous matrix around the starch molecules by disrupting the hydrogen bond among neighboring protein strands and via reducing interchain engaging forces. Such a matrix may also contribute to the increased chain mobility, flexibility and homogeneity of the water scattering in the sample [39]. In our study, the intensity ratio 1047/1022 cm^−1^ was increased after 5 days, implying a reduced amount of amorphous material, giving a more organized starch because retrogradation commenced. At first day, the lowest ratio was observed for 5% propylene glycol sample. There was not any significant difference between other samples. After 5 days, the ratios of the peak intensities at 1047 and 1022 cm^−1^ decreased with increasing of polyol level in model systems. Polyols incline to stabilize water–starch systems by becoming incorporated into the structure of the water that encloses the starch chain. This interaction of polyols with water creates the water “unfreezable,” resulting in a decrease of freezable water in the system and consequence of softening effect [40]. The highest and lowest ratios were associated to 1% glycerol and 5% propylene glycol samples. This reaction might be made happen by the smaller size of propylene glycol. It is able to go through the starch chains more simply and the crystallinity does not hinder this penetration [41]. These results are similar to those of earlier reports [42, 43].

**Table 2:** IR ratio of the absorbances 1047/1022 cm^−1^ for model systems.

Polyol	Level	Day 0	Day 5
Control	-	1.109^a^	1.321^a^
Glycerol	1	1.043^b^	1.267^b^
	5	1.038^bc^	1.189^c^
Sorbitol	1	1.037^bc^	1.158^d^
	5	1.030^c^	1.102^e^
Propylene glycol	1	1.034^bc^	1.073^f^
	5	0.998^d^	0.969^g^
SEM (±)		(0.0033)	(0.0045)

Each observation is a mean of three replicate experiments (n = 3).

Values in columns with different letters are significantly different (*P* ≤ 0.05).

SEM, standard error of the mean.

### 3.3 Effect of polyols on gluten

Stretch vibration band of N-H was evident at 3433 cm^−1^ for gluten (Fig. 1). The positions of this vibration band of N-H coincide with the band of the OH stretching which lead to development of envelopes with main bands at 3433 cm^−1^. Polyols addition resulted in increase of water captured in the model systems which was consistent with the increased bands at 1654 (OH bending) and 3433 cm^−1^ (OH stretching). In addition, the absorption bands between 1241–1472 cm^−1^ were possibly enhanced due to increase of polyols in the models. These bands are attributable to the C-O-O and C-N stretching and N-H bending (amide III) vibrations of gluten and inulin and C-OH stretching of polyols. Similarly, the bands at 1161, 1079 and 1022 cm^−1^ which are due to the C-O stretching mode of the C-OH groups of gluten, inulin and polyols [44, 45, 46], were increased as a result of polyols addition. Moreover, stretching vibrations of C-O-C and out of plane bending modes of C-H corresponding to the gluten and polyols were increased at 866 cm^−1^ with polyols addition in models [15]. Peaks within the 1500–1800 cm^−1^ area are related to Amide I and II signals of protein, carbonyl stretching modes of inulin and the O–H bending mode of water [47]. This region of the spectra is explored in more detail in the following paragraph using curve fitting routines.

**Fig. 3:**
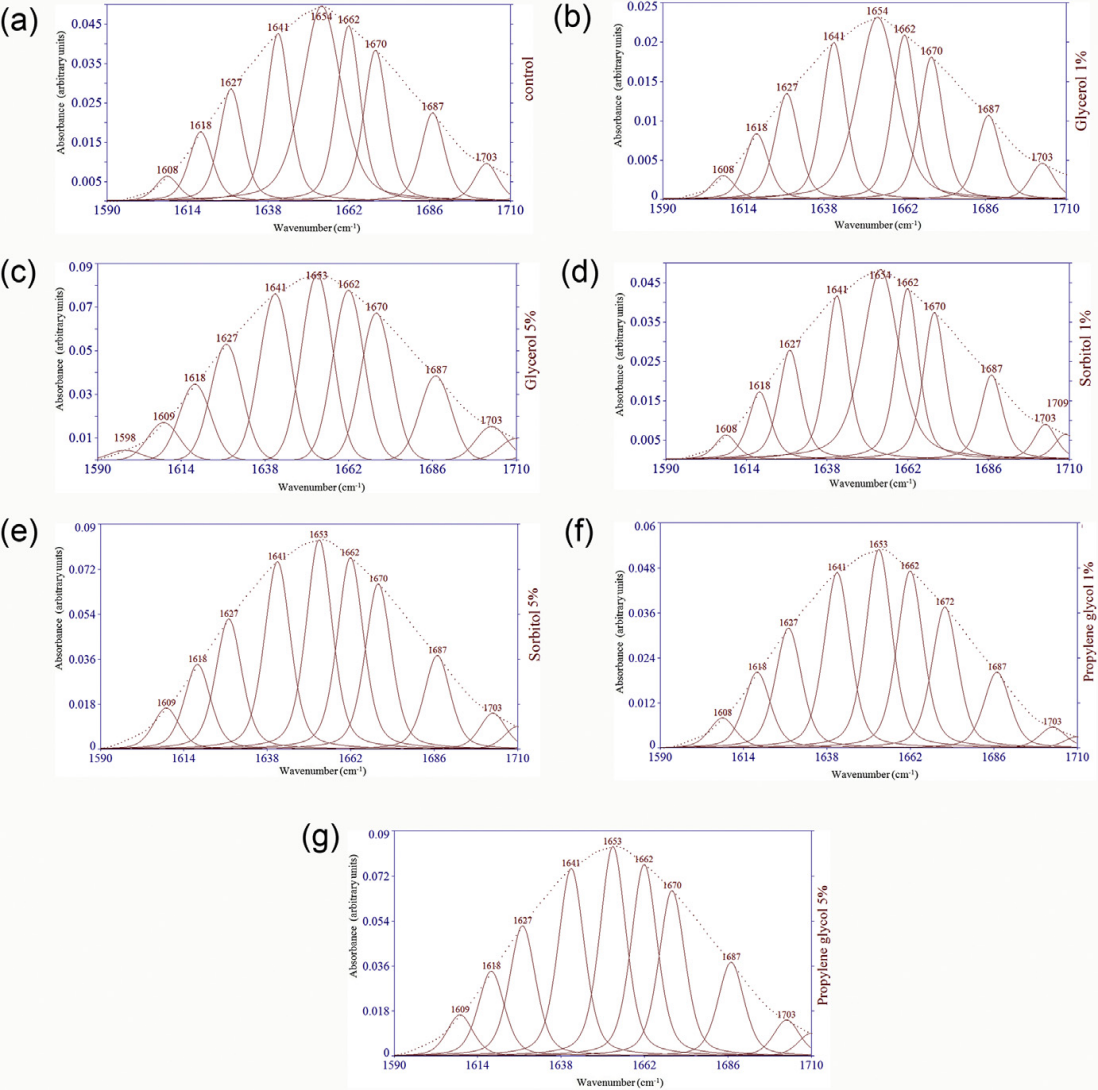
Curve fitted FTIR spectra for control (a) and models containing glycerol 1% (b), glycerol 5% (c), sorbitol 1% (d), sorbitol 5% (e), propylene glycol 1% (f) and propylene glycol 5% (g); Gaussian–Lorenzian mix function was used for curve fitting of the amide I band region (1600–1700 cm^−1^).

Amide I (1600-1700 cm^−1^) infrared spectra give a simple and consistent procedure for the reveal of the secondary structures of proteins in aqueous solution. The growth in the development of methods for examination of spectral data makes it easier to differentiate the individual components within the basically coincided amide I band outlines [16]. To explore the structure of the gluten proteins contributing to the Amide I bands, curve fitting techniques were used to the initial spectra of models throughout the area of 1600–1700 cm^−1^ (Fig. 3). All models had intense absorption bands at ∼1654 cm^−1^, which display α-helical structures [48]. The presence of α-helices is further confirmed by the attendance of Amide II band found at ∼1560 cm^−1^ [49]. The peak at ∼1670 cm^−1^ in the spectra specifies the occurrence of β-turns which initiated from the glutamine side chains of gluten. The absorptions at ∼1627 and 1641 cm^−1^ likely resulted from the intermolecular β-sheets [48]. As can be seen in Table 3, the most β-turns (32.19%) and intramolecular β-sheets (12.13%) were observed in the models containing 5% propylene glycol. The least content of α-helix was detected in the models of 5% propylene glycol, 5% sorbitol and 1% propylene glycol. The least amount of intermolecular β-sheets was identified in the models of 5% and 1% propylene glycol. Upon Gaussian–Lorenzian curve fitting, the relative contribution of peaks characteristic of β-turns and intramolecular β-sheets were almost increased when polyol proportion of models increased. While, content of intermolecular β-sheets and α-helix was almost decreased with increasing of polyols in the models. The supplementation of polyols to models probably altered the hydrogen bonds created between polypeptides. These hydrogen bonds are more distinctive in short chains like propylene glycol. Propylene glycol may interact through hydrogen bonds found between its hydroxyl groups and neighboring polypeptide chains. These interactions become more fragile with increasing of the length of the polyols. Glycerol is illustrated by three -OH groups and an asymmetric composition; therefore the interactions with polypeptide chains should be more complex, leading to a fewer organized network, with a different consequence in the secondary structures. β-spiral compositions can be considered as repeated β-turns and are recognized as one of the structural features relevant to the viscoelasticity of dough [48]. Anything that transforms the β-spiral structure into intermolecular β-sheet structure would negatively influence the quality of breadcrumb [16]. This behaviour increases in the number of junction zones and may have an impact on loaf volume. Such aggregated junction zones are likely to reveal a greater degree of rigidity and lack of flexibility compared to hydrated β-turns in terms of rheological properties. It eventually results in a decline in rheological properties such as extensibility and expansion capacity in the dough. These properties are important for the development of acceptable loaf volume and crumb structure in bread [16]. In agreement with Pourfarzad et al. [5], inulin supplement has an unfavorable effect on loaf volume of bread due to destruction of the β-spiral structure to intermolecular β-sheets. As revealed in the previous part, almost all of the β-turn and intramolecular β-sheet substances were decreased but intermolecular β-sheet and α-helix contents were increased by the addition of polyols to model systems. The decrease in the intermolecular hydrogen-bonded β-sheets might be due to the fact that the polyol molecules penetrate the sites in which neighboring molecules tightly associate via β-sheets. In the absence of the polyols, water molecules move more freely and can more easily approach gluten, causing increased intermolecular β-sheets which in turn stabilizes β-turns [24]. The loop-train theory developed by Belton (1999) briefly explains the phenomena observed during dough mixing and development that leads to the formation of glutenin polymers [50]. According to this theory, β-sheet secondary structures (trains) are naturally less elastic than β-turn secondary structures (loops) in gluten protein. Transition from β-turn to β-sheet secondary structures results in torque increasing and strain hardening during mixing. The progressive stretching and arrangement of the cross-linked gluten network during mixing eventually results in a threshold ratio of β-sheet to β-turn structure that is resistant to more deformation. Mixing further than this point results in the breakdown of the gluten network and subsequently decrease in mixing force. In summary, polyols could be used to reduce the undesirable effects of inulin on the quality parameters of dough and bread.

**Table 3:** Effect of polyols addition on the secondary structures content in models.

Polyol		Secondary structures			
Type	Level (%)	Intramolecular β sheet (%)	β turn (%)	α helix (%)	Intermolecular β sheet (%)
Control	0	9.55^e^	31.30^e^	19.56^a^	36.58^a^
Glycerol	1	11.40^d^	31.30^e^	19.33^b^	35.31^b^
	5	11.40^d^	31.34^d^	19.31^c^	35.31^b^
Sorbitol	1	11.40^d^	31.30^e^	19.16^d^	35.31^b^
	5	11.94^b^	32.07^c^	18.36^e^	34.43^c^
Propylene glycol	1	11.69^c^	32.14^b^	18.36^e^	34.30^d^
	5	12.13^a^	32.19^a^	18.36^e^	34.29^d^
Standard Error of Mean		±0.007	±0.006	±0.006	±0.005

Means with the same letters are not significantly different at 5% probability level.

## 4 Conclusion

The Fourier transform infrared spectroscopy was applied for screening of complex-formation and interactions between macromolecules of a bread model system fortified with inulin. The incorporation of polyols caused different changes in starch and gluten structures. The intensity ratio of 1047 cm^−1^ ⁄ 1022 cm^−1^ shows the extent of order in starch. This ratio was reduced with addition of polyols to models during 5 days storage, indicating the lower amount of starch retrogradation. At first day, the lowest ratio was observed for 5% propylene glycol sample. After 5 days, the highest and lowest ratios were related to 1% glycerol and 5% propylene glycol samples. Almost all the β-turn and intramolecular β-sheet contents were decreased while intermolecular β-sheet and α-helix contents were increased by the addition of polyols to model systems. The extent of these conversions was related to the type and concentration of added polyols. The most β-turns and intramolecular β-sheets were observed in the models containing 5% propylene glycol. The least content of α-helix was detected in the models of 5% propylene glycol, 5% sorbitol and 1% propylene glycol. The least amount of intermolecular β-sheets was identified in the models of 5% and 1% propylene glycol. In conclusion, the best quality and shelf life of bread fortified with inulin were obtained in samples containing 0.5% propylene glycol. This knowledge will considerably facilitate designing new formulas of the inulin blends as more useful for supplementation of bakery products. By eliminating negative quality and shelf life effects of the supplementation using polyols, the consumption of inulin-fortified bread will increase for the benefit of public health.

## Declarations

### Author contribution statement

Amir Pourfarzad, Zahra Ahmadian, Mohammad B. Habibi-Najafi: Conceived and designed the experiments; Performed the experiments; Analyzed and interpreted the data; Contributed reagents, materials, analysis tools or data; Wrote the paper.

### Funding statement

This work was supported by the Iran National Science Foundation (INSF) via the research project number 93043265.

### Competing interest statement

The authors declare no conflict of interest.

### Additional information

No additional information is available for this paper.
